# Deciphering the cellular source of tumor relapse identifies CD44 as a major therapeutic target in pancreatic adenocarcinoma

**DOI:** 10.18632/oncotarget.3510

**Published:** 2015-03-10

**Authors:** Maria Inés Molejon, Juan Ignacio Tellechea, Celine Loncle, Odile Gayet, Marine Gilabert, Pauline Duconseil, Maria Belen Lopez-Millan, Vincent Moutardier, Mohamed Gasmi, Stephane Garcia, Olivier Turrini, Mehdi Ouaissi, Flora Poizat, Nelson Dusetti, Juan Iovanna

**Affiliations:** ^1^ Centre de Recherche en Cancérologie de Marseille (CRCM), INSERM U1068, CNRS UMR 7258, Aix-Marseille Université and Institut Paoli-Calmettes, Parc Scientifique et Technologique de Luminy, Marseille, France; ^2^ Hôpital Nord, Marseille, France; ^3^ Institut Paoli-Calmettes, Marseille, France; ^4^ Hôpital de la Timone, Marseille, France

**Keywords:** pancreas cancer, xenograft, CD44, recurrence

## Abstract

It has been commonly found that in patients presenting Pancreatic Ductal Adenocarcinoma (PDAC), after a period of satisfactory response to standard treatments, the tumor becomes non-responsive and patient death quickly follows. This phenomenon is mainly due to the rapid and uncontrolled development of the residual tumor. The origin and biological characteristics of residual tumor cells in PDAC still remain unclear. In this work, using PDACs from patients, preserved as xenografts in nude mice, we demonstrated that a residual PDAC tumor originated from a small number of CD44+ cells present in the tumor. During PDAC relapse, proliferating CD44+ cells decrease expression of ZEB1, while overexpressing the MUC1 protein, and gain morphological and biological characteristics of differentiation. Also, we report that CD44+ cells, in primary and residual PDAC tumors, are part of a heterogeneous population, which includes variable numbers of CD133+ and EpCAM+ cells. We confirmed the propagation of CD44+ cells in samples from cases of human relapse, following standard PDAC treatment. Finally, using systemic administration of anti-CD44 antibodies *in vivo*, we demonstrated that CD44 is an efficient therapeutic target for treating tumor relapse, but not primary PDAC tumors. We conclude that CD44+ cells generate the relapsing tumor and, as such, are themselves promising therapeutic targets for treating patients with recurrent PDAC.

## INTRODUCTION

Pancreatic ductal adenocarcinoma (PDAC) is one of the deadliest cancers worldwide, due to early metastases and strong chemoresistance [1, 2]. This chemoresistance is due, in part, to the characteristic stromal composition of these cells, which acts as a mechanical barrier, and the subsequent reduced vascularization of the cellular environment, both of which interfere with the ability of drugs to reach the target cells [3]. Furthermore, biological, molecular and genetic features of pancreatic cancer cells, which impair drug entry into the cells, or affect cellular metabolism, may increase the chemoresistance of PDAC [4]. However, the variation in expression of these cancer cell properties generates diverse grades of PDAC resistance, each of which requires adapted treatment, which may in turn affect survival time for patients.

Clinically, PDAC disease progresses rapidly and causes patient death in the majority of cases, though some pancreatic tumors show a temporary objective response to treatment, followed by a systematic resistant period [5-7]. Resistant cancer cells are refractory to therapy and produce tumors that grow uninterrupted, ultimately causing rapid patient decline and death. Conversely, for sensitive tumors, two theories exist to explain PDAC recurrence. Firstly, it is thought that during the course of treatment, some cancer cells become progressively resistant to the antitumoral drugs, generating a gradually resistant tumor. Secondly, it has been proposed that the residual tumor starts from a cancer cell population that is naturally not targeted by the anticancer agents. Moreover, the possibility that residual tumors may result from a combination of both a selection of resistant cells along with an expansion of a marginal population of naturally insensitive cells, cannot be excluded.

It is noteworthy that current chemotherapeutic strategies are based on targeting rapidly dividing cells. However, most tumors, including PDACs, possess a specific, small, non-replicative cell population, which may be referred to as cancer stem cells (CSCs) [8]. These CSCs are thought to be insensitive to anticancer treatments and could be the source of residual tumors [9]. Current consensus describes CSCs as cells within a tumor, which are able to self-renew, and produce a heterogeneous lineage of cancer cells which are represented in the tumor [10]. The stem cell hypothesis has recently been explored in pancreatic cancer [11]. The prospective identification of CSC populations from various tumors of epithelial origin, including the breast, colon and prostate, has been undertaken [12-15]. *Ex vivo*, the population of CSCs can be identified using various surface markers, the most common of which are a combination of EpCAM, CD44 and CD24 or the single marker CD133 [8, 11, 16]. Evidence supporting the chemoresistance of the stem-like cells in epithelial cell lines and xenogeneic tumor-derived cells has been reported [12, 17-21].

A residual tumor can be defined as a small tumor mass that grows after an incomplete response to chemotherapy treatment of the original tumor, and is the origin of tumor relapse, which ultimately results in patient death. Hence, residual tumor cells are the key factor responsible for tumor recurrence. However, the origin and biological characteristics of PDAC residual tumor cells remain unclear. Therefore, theoretically, primary and residual tumors could consist of cells with different biological characteristics, which may suggest that treatments for primary and relapsing tumors should be based on different therapeutic strategies. In this paper, using PDACs from patients, preserved as xenografts in nude mice, we report that cells expressing the CD44 marker are at the source of the residual tumor, following standard antitumoral treatment. We found that these cells proliferate in the tumor and simultaneously lose dedifferentiation markers and gain markers associated with differentiation. Finally, we also found that targeting residual tumor cells with a specific antibody makes it possible to efficiently block tumor growth, although the same treatment was almost entirely ineffective for treating primary PDAC tumors. In conclusion, in this paper we propose that treatment of residual tumors should be revised and adapted to their cell specific biology.

## RESULTS

### The heterogeneous responses of PDAC-derived xenografts to gemcitabine treatment

**Figure 1:**
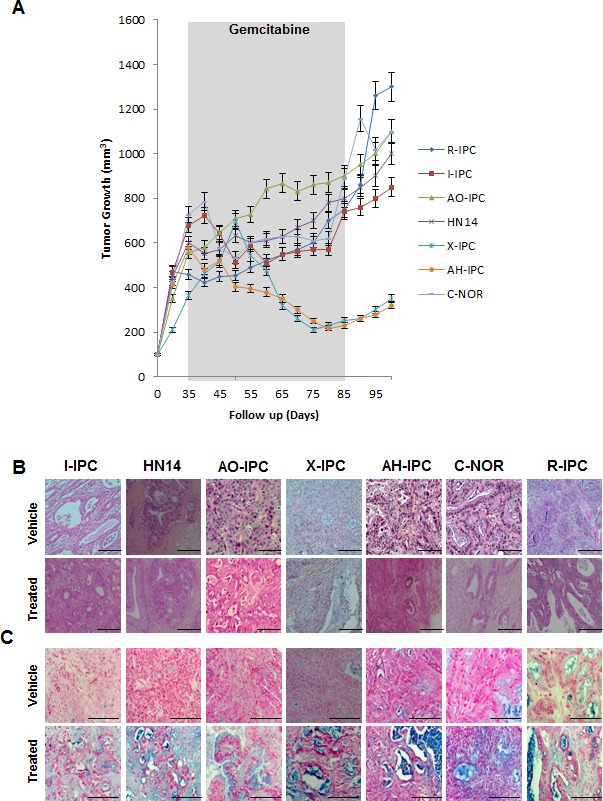
Heterogeneous response to gemcitabine treatment (A) Mice bearing primary xenografts were treated with 100 mg/kg gemcitabine, and were measured weekly for changes in tumor volume. The length of the treatment is marked in gray. (B) Various histological features were detected by H&E staining of PDAC-vehicle treated tumors (upper panel) and gemcitabine-treated tumors (bottom panel). (C) PDXs Alcian Blue staining stains acid mucosubstances and acetic mucins. Scale bar represents 100 and 50 μm. Error bars ± SEM; n=3 per group.

Seven patient-derived xenografts (PDX) were selected for study. Clinical and histopathological characteristics of the patients and the PDAC tumors are presented in Supplemental Table S2 and Figure S1. As shown in Figure 1A, X-IPC and AH-IPC tumors were highly sensitive, while I-IPC and C-NOR tumors were moderately sensitive to therapy. Meanwhile AO-IPC, HN14 and R-IPC tumors were highly resistant to gemcitabine treatment as shown in Figure 1A. However, after treatment withdrawal, both moderately sensitive and sensitive PDXs, started to re-grow (Figure 1A). Surprisingly, histological analysis of the PDX tissues revealed that after treatment, all tumors presented with morphological features of improved differentiation, with a gain in glandular formation, a well polarized phenotype (see Figure 1B) and a strong production of mucus, as demonstrated by alcian blue staining (Figure 1C). These results allow us to conclude that following gemcitabine treatment, the PDAC tumor cell phenotype alters, to become more differentiated.

### Cells expressing CSC-associated markers are enriched in relapsing tumors following gemcitabine treatment

**Figure 2:**
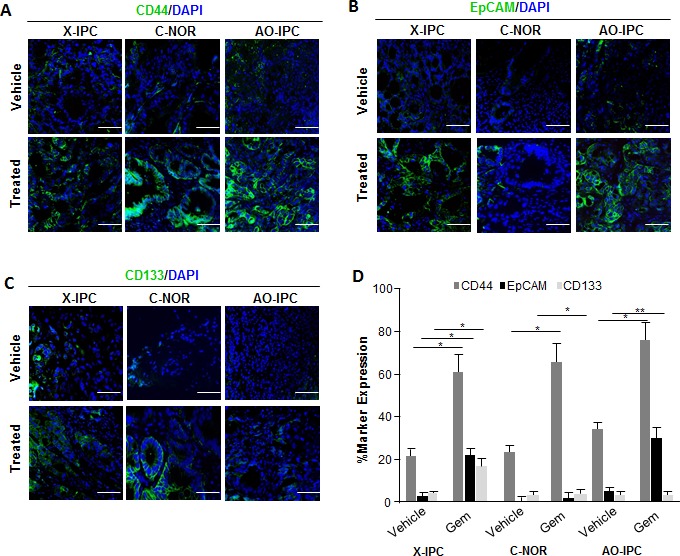
Expression of Cancer Stem Cell (CSC) markers in PDXs Immunofluorescent labeling of (A) CD44-FITC (green) and 4′,6-diamidino-2-phenylindole (DAPI), (blue); (B) EpCAM-FITC (green); DAPI (blue) and (C) CD133-FITC (green); DAPI (blue) in PDXs upon vehicle or gemcitabine treatment, show the distribution of each CSC marker in tumor tissues. (D) Quantification of the number of positive cells in each PDX. Scale bar represents 50 μm. Error bars ± SEM; n=3 per group. *P<0.05, **P<0.001 compared to vehicle treatment.

Considering that CSCs represent the only cell population with tumor-initiating potential, we hypothesized that these cells, which seem to play a crucial role in treatment resistance, are associated with the phenotypic transformation described above. These cells are capable of resisting toxicity by drugs, such as gemcitabine, that target highly proliferative cells, since they enter into the cell cycle less frequently and only divide in response to certain stimuli, which have yet to be fully identified. We sought to investigate the potential involvement of CSCs in driving residual tumor growth following gemcitabine treatment. We used immunohistochemistry to analyze the expression of some CSC-associated cell surface markers on these tumors. We selected PDXs which were either sensitive to, or moderately or highly resistant to gemcitabine therapy (X-IPC, C-NOR and AO-IPC, respectively). Those PDXs reaching a volume of 400 mm^3^ received either vehicle or 100 mg/kg gemcitabine, twice weekly. Immunofluorescence analysis revealed that the percentage of CD44+, EpCAM+ and CD133+ cells in gemcitabine-treated tumors increased systematically 3-4 folds when compared to vehicle-treated tumors. As shown in Figure 2A, CD44 expression was found in 22±2.8%, 25±2.3% and 30±4.4% of vehicle-treated tumors, while its expression increased to 59±7.0%, 65±7.2% and 73±7.9% in gemcitabine-treated X-IPC, C-NOR and AO-IPC, respectively. Similarly, EpCAM was expressed in 3.6±1.0%, 0.2±0.7% and 4.3±0.8% of vehicle-treated tumors and increased to 24±2.8%, 1.2±1.3% and 31±3.3% in gemcitabine-treated X-IPC, C-NOR and AO-IPC, respectively (Figure 2B). Finally, CD133 was expressed in 5.0±0.5%, 1.0±0.9% and 5.1±0.8% of vehicle-treated tumors, while in gemcitabine-treated tumors its expression was found in 18±2.7%, 3.2±1.2% and 5.3±1.2% of X-IPC, C-NOR and AO-IPC, respectively (Figure 2C). Quantification is shown in Figure 2D. Thus, these data strongly suggest that gemcitabine treatment may select a highly enriched population of cells expressing CSC-associated markers *in vivo*. Interestingly, CD44+ cells are the most abundant population, when compared with the expression of EpCAM and CD133. In addition, this selection is independent of the degree of gemcitabine chemosensitivity of each tumor.

### CD44+ cells present phenotypical characteristics of differentiation, and proliferate in relapsing PDAC tumors

**Figure 3:**
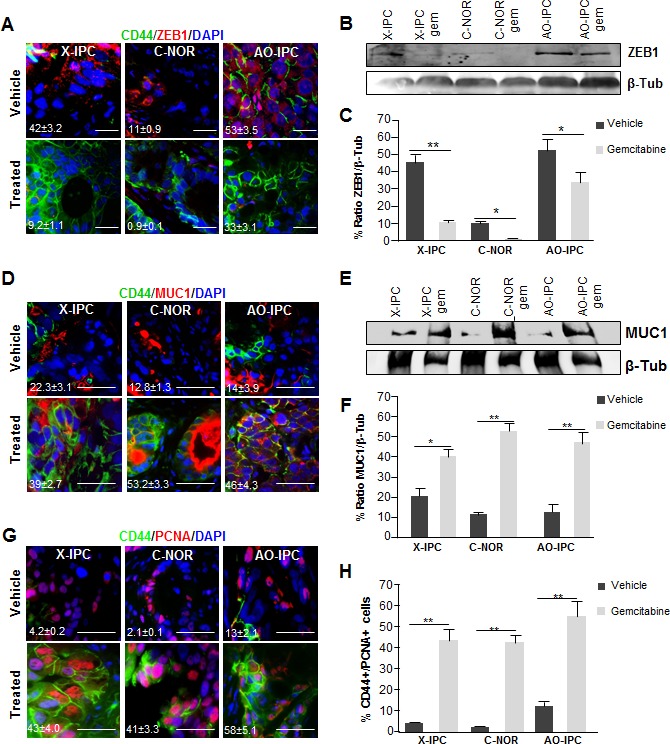
Deregulation of differentiation markers in PDXs after chemotherapy (A) Immunofluorescent analysis of CD44 (green), ZEB1 (red) and DAPI (blue) in three PDX samples treated with vehicle (upper panel) or with gemcitabine (bottom panel). (B) WB analysis for ZEB1 in protein lysates from tumor samples upon vehicle or gemcitabine treatment. (C) Quantification of ZEB1 protein expression. (D) Co-localization analysis of CD44 (green), MUC1 (red) and DAPI (blue) in PDX samples treated with vehicle (upper panel) or with gemcitabine (bottom panel). (E) WB analysis for MUC1 in protein lysates from tumor samples treated with vehicle or with gemcitabine. (F) Quantification of the expression of the MUC1 protein in tumor samples. (G) Immunofluorescent labeling of PCNA (red), CD44 (green) and DAPI (blue) in PDXs vehicle or treated with gemcitabine. The CD44+/PCNA+ ratio is shown on the left side (H). Scale bar represents 10 μm. Error bars ± SEM; n=3 per group. *P<0.05, **P<0.001 compared to vehicle treatment.

To characterize the evolution of CD44-expressing cells, we investigated the expression of cell differentiation-associated markers. To this end, we used immunofluorescence to analyze the expression of the ZEB1 protein, which has been associated with poor differentiation, and CD44. We found a significant decrease of ZEB1 expression after gemcitabine treatment. Remarkably, we detected ZEB1 in 42±3.2%, 11±0.9% and 53±3.5% of cells in vehicle-treated PDXs whereas its expression decreased to 9.2±1.1%, 0.9±0.1% and 33±3.1% after gemcitabine treatment in X-IPC, C-NOR and AO-IPC PDXs, respectively (Figure 3A). ZEB1 frequently colocates with CD44 in vehicle-treated PDXs but rarely in gemcitabine-treated PDXs. The decreased expression of ZEB1 after gemcitabine treatment was confirmed by western blot analysis (Figure 3B and 3C). Furthermore, we evaluated MUC1 expression, which, conversely, is strongly associated with cellular differentiation. As illustrated in Figure 3D, though some cells of the vehicle-treated PDXs expressed MUC1, but never colocalizes with CD44+ cells. However, after gemcitabine treatment we found that the majority of the CD44+ residual cells expressed MUC1 (Figure 3D and F). Western blot analysis confirmed that MUC1 expression dramatically increased in gemcitabine-treated tumors, when compared to vehicle-treated samples (Figure 3E).

The increased number of CD44+ cells after gemcitabine treatment of PDXs may be attributed to the ability of these cells to proliferate, and the expression of the CD44 marker in resistant cells, in the absence of cell proliferation. Therefore, we evaluated the capacity of CD44+ cells to proliferate by measuring the expression of the PCNA proliferation-associated marker. Notably, upon gemcitabine treatment *in vivo*, surviving cells, bearing the CD44+ phenotype, re-enter the cell cycle en masse, as demonstrated by the significant increase in PCNA staining (Figure 3G and H). The proportion of PCNA positively stained nuclei in CD44+ cells, after gemcitabine treatment, increased from 4.2±0.2%, 2.1±0.1% and 13±2.1% to 43±4.0% 41±3.3% 58±5.1% in X-IPC, C-NOR and AO-IPC, respectively. In summary, these results strongly indicate that CD44+ cells have a limited proliferation ratio in vehicle-treated cells but, conversely, the recurrent tumor develops mainly from CD44+ cells, since they show a high proliferative index, coupled with a loss of the epithelial to mesenchymal transition (EMT) marker ZEB1, and an increased expression of the differentiation associated protein MUC1.

### Expression of CSC-associated markers does not predict the chemosensitivity of PDAC-derived cells *in vitro*

**Figure 4:**
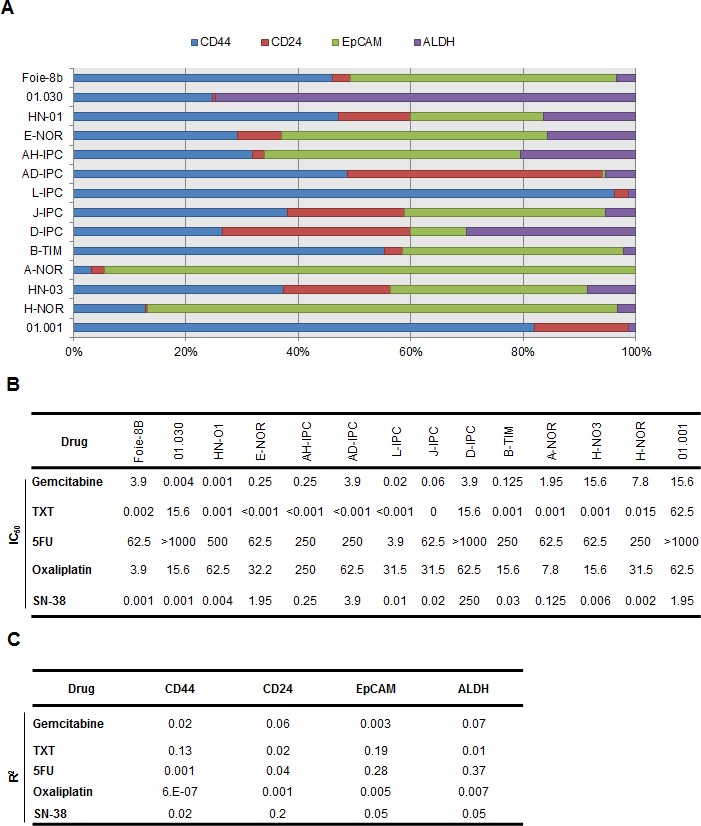
CSC-associated marker expression *in vitro* and its relationship to chemosensitivity (A) Flow cytometry was performed to identify quadruple staining for CD44-APC, EpCAM-VioBlue, CD24-PE and ALDH-FITC in fourteen primary cell-derived xenografts (n = 3). (B) Each cell line was treated with increasing concentrations (from 0 to 1000 μM) of Gemcitabine, Docetaxel (TXT), 5-Fluouracil (5FU), Oxaliplatin and the active metabolite of Irinotecan known as SN-38. The rate of cell survival was measured after 72 h of treatment. A sensitivity profile was obtained for each drug and the IC_50_ data is presented in the table. (C) Linear regression analysis to assess the level of expression of each CSC-associated marker and IC_50_ corresponding to each drug, was performed. N=3 per group.

The association between chemoresistance and CSC marker expression *in vitro* has been proposed, though not yet confirmed. We developed an approach to study whether the expression of some CSC-associated markers is predictive of chemosensitivity in PDAC-derived cells. We established an independent set of 14 primary cultures, from PDXs obtained from patients with PDAC. The expressions of CD44, CD24, EpCAM and ALDH activity, all of which are CSC markers, were measured by flow cytometry analysis. Results are presented in Figure 4A. As shown, CD44 expression varied from 3.2 to 96.2%, CD24 from 0.5 to 45.3%, EpCAM from 0 to 94.2% and ALDH activity ranged from 0 to 74.5% in the PDX-derived cells (see Table S3). We then determined the IC_50_ for the 5 most commonly used drugs in the treatment of patients with PDAC, namely gemcitabine, 5FU, oxaliplatin, docetaxel (TXT) and SN-38 in these PDX-derived cells (Figure 4B and Figure S2) and analyzed its relationship with the expression of CSC markers. Importantly, no correlation was found between the sensitivity of the PDX-derived cells to each drug, and the amount of cells expressing CSC-associated markers individually or in combination (Figure 4C and Figure S3). We conclude that the number of cells expressing CSC markers in a population of cells derived from a PDX, does not predict its sensitivity to the more frequently used PDAC treatments.

### CSC putative CD44+ cells, present in relapsing tumors, are sensitive to anticancer drugs *in vitro*

**Figure 5:**
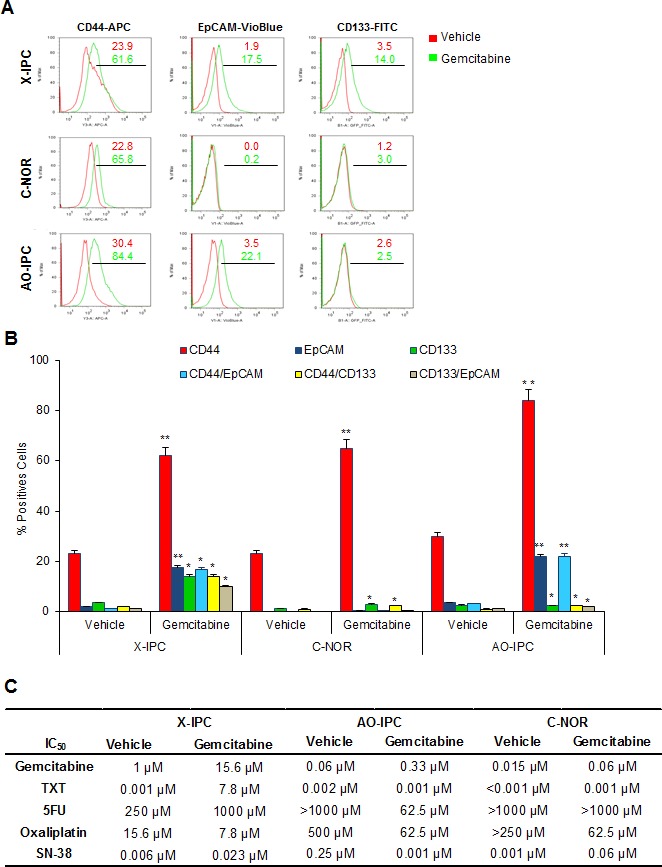
Sensitivity of PDX-derived cells *in vivo* (A) Representative flow cytometry plots for CD44-APC, EpCAM-VioBlue and CD133-FITC in three PDXs treated with vehicle or 100 mg/kg gemcitabine. (B) Quantification of flow cytometry analysis performed in A. (C) Vehicle or Gemcitabine PDX-derived cells were treated with increasing concentrations of Gemcitabine, Docetaxel (TXT), 5-Fluouracil (5FU), Oxaliplatin and the active metabolite of Irinotecan known as SN-38. The rate of cell survival was measured after 72 h of treatment. Error bars ± SEM; n=3 per group. *P<0.05, **P<0.001 compared to vehicle treatment.

Since the expression of CSC markers does not predict the responsiveness to the anticancer treatments, we further studied whether CSC-like cells, present in the residual tumors, are insensitive to the most commonly used PDAC chemotherapeutics. First, we used flow cytometry to measure the percentage of CD44+ cells in primary cultures obtained from X-IPC, C-NOR and AO-IPC PDXs, treated with vehicle or with gemcitabine. We found a significant difference in the proportion of CD44+ cells, from 23.9% to 61.6% for X-IPC, from 22.8% to 65.8% for C-NOR and from 30.4% to 84.4% for AO-IPC, between vehicle and gemcitabine treated PDXs respectively. We also measured expression of EpCAM and CD133 in these primary cultures and found a wide variation between tumors from 0% to 3.5% in vehicle-treated cells and from 0.2% to 22.1% in gemcitabine-treated tumors for EpCAM. The values for CD133 were 1.2% to 3.5% for vehicle-treated cells and from 2.5% to 14.0% for gemcitabine-treated cells (Figure 5A, B). More importantly, we found that almost all of the EpCAM+ and CD133+ cells were included in the CD44+ cell population (Figure S4). Altogether these data allow us to suggest that residual tumors originate mainly from a population of CD44+ cells in PDAC, and that CD44+ cells are a heterogeneous population. We then examined the impact of treatment with increasing doses of gemcitabine, docetaxel, 5FU, oxaliplatin and SN-38 in primary cultured cells, obtained from PDXs, treated with vehicle or with gemcitabine. As expected, treatment with increasing concentration of gemcitabine showed a higher resistance to the drug, with a IC_50_ ranging from 1 to 15.6 μM, 0.06 to 0.33 μM and 0.015 to 0.06 μM for X-IPC, C-NOR and AO-IPC vehicle or gemcitabine-treated cells, respectively, as showed in Figure 5C. Unexpectedly, chemosensitivity to docetaxel, 5FU, oxaliplatin and SN-38 varied one cell to another for PDX-derived cell population. It should be: Unexpectedly, chemosensitivity to docetaxel, 5FU, oxaliplatin and SN-38 varied from one PDX-derived cell population to another, according to tumor and drug utilized as shown in Figure 5C. These results allow us to conclude that a higher expression of putative CSCs (CD44+) in a residual tumor does not predict the sensitivity to chemotherapeutic treatments *in vitro*.

### CD44 is an efficient therapeutic target for treating PDAC relapse

**Figure 6:**
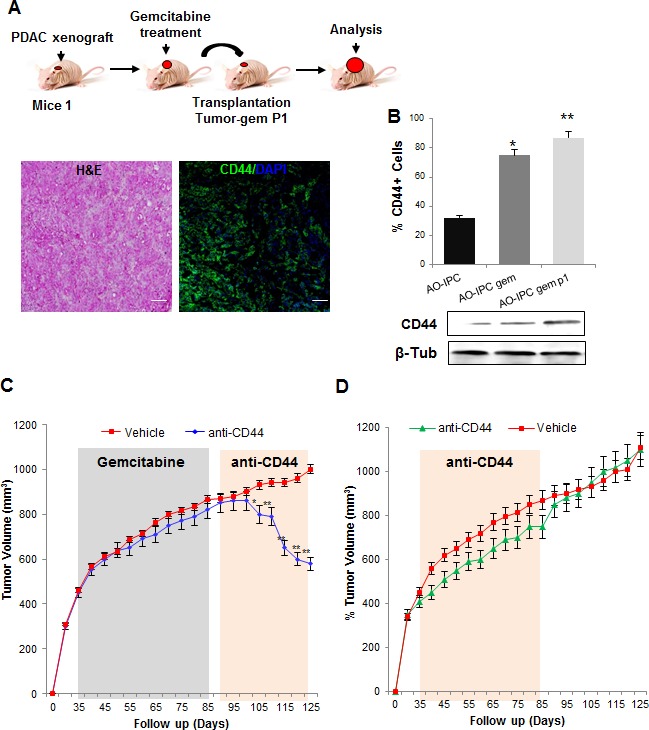
Depletion of CD44 for PDAC relapse treatment (A) Gemcitabine treated tumors were transplanted into new mice and allowed to continue to grow (tumor-gem P1), and CD44 expression was evaluated by immunofluorescence and by western blot. (B) Quantification of CD44 expression is shown. (C) AO-IPC xenografts treated with vehicle or gemcitabine (100 mg/kg, biweekly, from days 35 to 85) were then treated with anti-CD44 mAb 200 μg/mice biweekly and tumor volume was monitored weekly (mm^3^). (D) AO-IPC xenografts were treated with vehicle or anti-CD44 (200 μg/mice biweekly) from days 35 to 85. Scale bar represents 100 μm. Error bars ± SEM; n=3 per group. *P<0.05, **P<0.001 compared to vehicle samples.

CD44 is used in other cancers as an efficient therapeutic target [22]. Due to its increased expression in PDAC residual tumors, CD44 may be a viable therapeutic target to treat this disease. We therefore evaluated if, during long-term treatments with chemotherapeutic agents, CD44+ cells continued to accumulate. The PDXs were treated for one cycle of treatment with gemcitabine followed by transplantation of the relapsed tumors to other mice (Figure 6A). After sufficient tumor regrowth, we evaluated CD44 expression by immunofluorescence. We found that almost all cells were positive for CD44 staining, which indicated that relapsing tumors are composed mainly, if not exclusively, of CD44+ cells (Figure 6B). Consequently, because CD44 is highly expressed in relapsing PDACs, we evaluated the potential of using the anti-CD44 mAb to treat PDAC relapse in xenografts. Xenografts were transplanted to mice and treated by a cycle of gemcitabine as described in Figure 6A and mice bearing residual tumors were depleted of CD44 by systemic injection of the anti-CD44 mAb (200 μg/mice, twice weekly) in gemcitabine resistant-derived PDXs. As shown in Figure 6C, treatment with the anti-CD44 mAb significantly reduced tumor volume to the half (450 mm^3^ ± 6.2). However, when we injected the anti-CD44 antibody into mice bearing a gemcitabine-untreated PDX, almost no effect on tumor growth was found (Figure 6D) indicating the futility of targeting CD44 in primary non-residual tumors. Altogether, these data strongly suggest that relapsing PDACs are mainly formed from CD44+ cells, which could be a promising therapeutic target.

**Figure 7:**
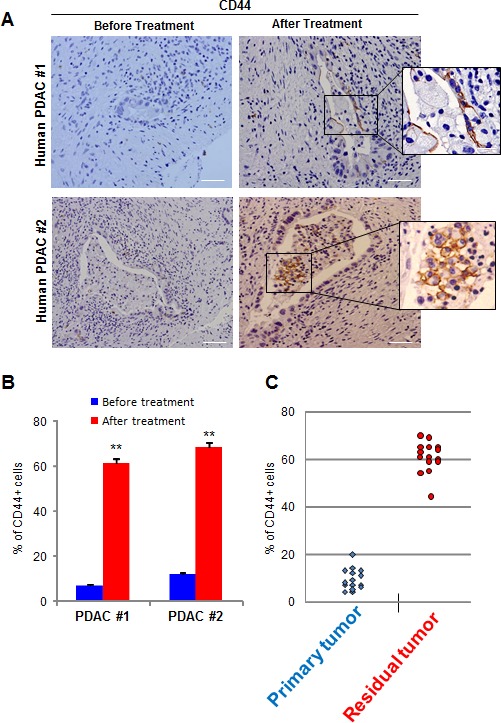
CD44 expression in human samples (A) CD44 expression was evaluated in human PDACs before and after chemotherapy. A magnification is shown on the right side of the figure. (B) Quantification of CD44-positive cells from. (C) Quantification of CD44-positive cells from 15 non-treated patients (Primary Tumor) and 15 chemotherapy treated patients (Residual Tumor) is shown. Scale bar represents 50 μm. Error bars ± SEM; n=15 per group. **P<0.001 compared to samples before treatment.

Finally, we studied CD44 expression in human PDACs. We collected 4 PDAC samples from the surgery of 2 patients who were deemed non-resectable after their cancers were found to be locally advanced. These patients underwent standard gemcitabine-based treatment, which allowed subsequent successful resection of their tumors, from which we obtained further PDAC samples. Therefore these samples allowed us the rare opportunity to compare PDAC samples from the same patients, before and after chemotherapy. Immunohistochemical analysis of CD44 expression showed that CD44+ cells were almost undetectable in tumorigenic cells from pretreated samples, whereas these cells were dominant after gemcitabine administration, as showed in Figure 7A. We then evaluated an additional set of 15 surgical samples from non-treated patients and 15 samples which were obtained after gemcitabine-based chemotherapy, and found similar results, as showed in Figure 7C. The findings obtained from human specimens collectively support our xenograft results, suggesting that CD44 could be an efficient therapeutic target for the treatment of residual but not primary human PDAC.

## DISCUSSION

Establishing the mechanism of tumor relapse, following chemotherapeutic treatment is very difficult, especially because of inter- and intra-tumoral heterogeneity. Here we show, by using direct xenografting from human PDACs, that, following robust treatment with gemcitabine, relapsing tumors are formed mainly from CD44+ cells. Therefore this indicates that CD44 is the most promising target for treating PDAC tumors after first-line therapy. We also observed that, as a consequence of chemotherapy treatment, while proliferating CD44+ cells lose the expression of ZEB1 [23] they gain expression of the differentiation-associated marker the MUC1 protein [24] and acquire morphological characteristics of a more differentiated tumor, suggesting a loss of the EMT and favoring the Mesenchimal to Ephitelial Transition (MET). In addition, we demonstrated that CD44+ cells consist of a heterogeneous population, since varying numbers of CD133+ and EpCAM+ cells were included in the population. Finally, we also found an efficient response to anti-CD44 in the treatment of relapsing but not primary PDAC tumors.

It is frequently observed in PDAC patients, that after a period of relatively satisfactory response to standard treatments, including a significant tumor volume decrease, the tumor becomes non-responsive and patient death quickly follows. This phenomenon is mainly due to the rapid and uncontrolled development of the residual tumor. In fact, the residual tumor is a tumor within another tumor, which has its own biological characteristics. During the time in which the standard treatment is effective, sensitive cells are eliminated but resistant and CSC-like cells remain. In addition, their coexistence allows the regulation of one another and the stromal compartment. After this period of responsiveness to therapy, the feedback control mechanism governed by the sensitive cells is lost, thus allowing greater access for the resistant cells to oxygen and nutrients. Furthermore, these cells have increased exposure to the factors necessary for tumor relapse, including CXCL12, through its receptors CXCR4 and CXCR7 [25], IL6 through the Jak2/Stat3 pathway [26, 27], EGF, FGF and IGF [28, 29], among others. This facilitates the rapid proliferation, and subsequent patient decline commonly seen, after standard treatments which do not target residual cells. CD44+ cells have been reported to be involved in the recurrence of several tumor types [30, 31] including PDACs after radiotherapy [32]. In this paper we present strong data regarding the origin of the relapsing PDAC, after treatment with the most commonly utilized anticancer drug. In addition, we were able to demonstrate that relapsing tumors are sensitive to anti-CD44 treatment, thereby supporting the use of this strategy as a possible treatment for PDAC at this clinically challenging phase. Interestingly, the FDA-approved humanized anti-CD44 mAb (RG7356) shows very promising results for treating some hematological malignant diseases [33, 34], which indicates that a clinical trial for treating patients with recurrent PDAC could easily be implemented.

However, definitive confirmation of CSCs as the origin of residual tumors has yet to be achieved, not least because a formal CSC classification system needs to be developed in PDAC but also elsewhere. In fact, even the characteristics of the PDAC-CSC population need to be established, since several putative markers such as CD44+, CD133+, CD24+, EpCAM+ and ALDH1 activity, alone or in combination, have been used to define CSC cells depending on author interpretation [35, 36]. Moreover, the choice of cancer model employed in a study may present challenges, since almost all studies have been performed on well-established cell lines, xenografted to produce tumors, or in cell culture. We used PDX samples which had the advantage of originating from primary tumors, with very low numbers of passages and therefore maintained the original structure and biological characteristics [37]. Under these experimental conditions, we found a significant variation in the number of cells expressing putative CSC markers as shown in Figure 2 and 4. However, the most represented cell type within PDAC tumors is CD44+, and notably, CD133+ and EpCAM+ cells are included within this population. Importantly, in relapsing tumors, the CD44+ cell volume expands, and CD133+ and EpCAM+ cells proportionally proliferate, indicating that the CD44+ cell population is heterogeneous within primary PDACs and relapsing tumors. The relapsing tumor has a larger proportion of CD44+ cells, than of CD133+ or EpCAM+ cells, making CD44 the most suitable therapeutic target. In this way, the utilization of anti-CD44 for treating residual tumors yields promising results. Moreover, in PDAC samples from treated patients we were able to demonstrate that CD44+ cells became the predominant population, whereas in pre-treatment tumors these cells are fewer in number, further supporting the use of anti-CD44 for treatment of recurrent tumors as a promising strategy. We found that PDACs from patients before chemotherapy showed a low number of CD44+ cells, whereas after chemotherapy residual tumors consisted almost entirely of CD44+ cells, as showed in Figure 7B and 7C. From our point of view, and based on the present data, we therefore assume that PDAC residual tumors originate from CD44+ cells rather than CSC cells, particularly because whether CD44+ cells may be classified as CSCs remains to be determined.

Although it has been previously suggested that chemosensitivity could be defined by the amount of CSC markers, this theory proved inconsistent. Although previous work reported that CD133+-xenografted cells are more resistant than CD133− cells to gemcitabine treatment [8], we found that cells expressing CSC markers were not particularly more resistant to a standard treatment than the other cells within the tumor, as presented in Figure 4 and 5. In fact, after strong enrichment of cells expressing CSC markers in residual tumors treated with gemcitabine, we observed, as expected, an increased IC_50_ to gemcitabine, probably due to clonal selection. However, in these cells the resistance to 5FU, oxaliplatin, SN-38 and docetaxel appeared to be PDX− and drug-dependent, as showed in Figure 5. These results suggest that CSCs could be more resistant to drugs targeting proliferation-dependent factors, but that proliferating CSC-like cells behave unpredictably in terms of chemosensitivity.

**Figure 8:**
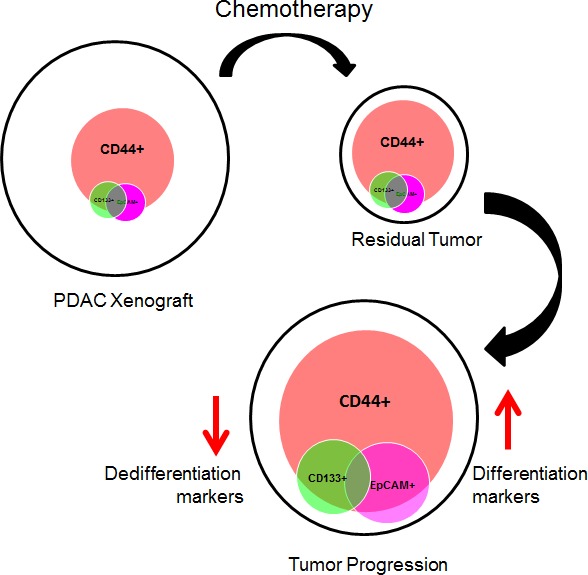
Schematic representation of the proposed model

A sensitive tumor probably predominately consists of drug sensitive cells, whereas a resistant tumor is formed chiefly of resistant cells. This is known as the intra-tumor heterogeneity [38, 39]. Also, a tumor could be resistant to one particular drug, but sensitive to another. This is why a tumor may initially reduce in volume after first-line therapy and then progress and ultimately cause patient death. Regarding CD44+ cell accumulation, our findings showed that this increase occurs in both gemcitabine-resistant and sensitive tumors, probably due to clonal selection, which also includes CD44+ cells, as mentioned above. In this study, we observed that following gemcitabine treatments, the heterogeneous population of CD44+ cells is therapy-resistant, and has unique characteristics such as loss of the expression of the EMT marker ZEB1 and increased expression of the differentiation marker MUC1, along with elevated mucus production and a dramatic gain of differentiation characteristics. In addition, these CD44+ relapsing cells have the capacity to proliferate, suggesting that these cells generate a relapsing tumor, with a more differentiated phenotype (Figure 8). The fact that relapsing tumors present biological characteristics of differentiation is not surprising, as in cases of small-cell lung cancer, which become resistant to chemotherapy, an increased expression of differentiation markers has previously been reported [20, 21, 40-42]. Finally, to evaluate if this is a gemcitabine-dependent mechanism, we quantified CD44+ cells in docetaxel-treated tumors and we found a very close response to gemcitabine-treated xenografts, suggesting that this mechanism is not exclusively associated to the gemcitabine treatment.

In summary, we present data demonstrating that relapsing PDAC tumors originate from the expansion of CD44+ cells. These CD44+ proliferating cells decrease the expression of a dedifferentiation marker, and gain morphological and biological characteristics of differentiation. Also, we report that CD44+ cells are a heterogeneous population that includes variable amounts of CD133+ and EpCAM+ cells. Based on these data, we demonstrated *in vivo* that CD44 is an efficient target for treating PDAC relapse, but not primary PDAC tumors.

## METHODS

### Tumor samples

Patients were recruited for this study under the Paoli Calmettes Institute clinical trial number 2011-A01439-32. After patient's informed consent had been obtained, excess tissue samples from resected PDACs were collected for xenograft procedures. Briefly, excess tumor tissue samples, obtained during routine resections, performed by surgeons, and not required for clinical diagnoses, were subsequently implanted into immunocompromised mice. Patient anonymity was maintained by removing any information which identified, or could lead to the identification of the patient.

### Animal experiments

All animal experiments were conducted in accordance with institutional guidelines and were approved by the “Plateforme de Stabulation et d'Expérimentation Animale” (PSEA, Scientific Park of Luminy, Marseille). Briefly, a total number of 7 human PDAC xenografts were established (Table 1). Tumor specimens (100 mm^3^), from resected PDAC patients, were mixed with Matrigel (BD Biosciences) and implanted subcutaneously on the upper right flank of 5- to 6-week-old nude mice (Swiss Nude Mouse Crl: NU(lco)-Foxn1nu, Charles River Laboratories) Tumor size and body weights of all animals were measured weekly. Subcutaneous tumor measurements were undertaken using calipers and values were calculated as (length × width^2^)/2. Gemcitabine (Lilly) treatment was administered twice weekly (100 mg/kg i.p.) until the tumor size was reduced by half or until the end of an 80 day experimental period. Following treatment, the tumor was allowed to grow for 15 additional days. For anti-CD44 treatment, anesthetized mice were administered purified whole mAbs against CD44 or nonspecific IgG (vehicle), intravenously (200 μg/animal), twice weekly, for 35 days after gemcitabine therapy [22].

### Histology and immunohistochemistry

Tumoral sections were paraffin embedded and hematoxylin and eosin (H&E), alcian blue, immunohistochemistry and immunofluorescence staining procedures were performed using standard protocols. Sections (5μm) were probed with primary antibodies: CD44 and EpCAM (BioLegends), CD133 (Merck Millipore), ZEB1 (Santa Cruz), MUC1 (Abcam) and PCNA (DAKO). Alexa Flour 488 and 594 (Invitrogen) were used as secondary antibodies. CD44 was probed on paraffin embedded human PDAC samples using DAKO EnVision+System-HRP (DAB) for mouse primary antibodies. For immunofluorescence, samples were mounted in ProLong Antifade Reagent with DAPI (Invitrogen) and examined with an Eclipse 90i Nikon microscope (Nikon Instruments Europe B.V., Champigny-sur-Marne, France).

### Immunoblotting

Protein extraction was performed, on ice, using total protein extraction buffer: 50 mM HEPES (pH 7.5), 150 mM NaCl, 20% SDS, 1 mM EDTA, 1 mM EGTA, 10% glycerol, 1% Triton, 25 mM NaF, 10 mM ZnCl_2_ and 50 mM DTT. Before lysis, protease inhibitor cocktail at 1:200 (Sigma-Aldrich; NUPR1340), 500 mM PMSF, 1 mM sodium orthovanadate and 1 mM β-glycerophosphate were added. Protein concentrations were measured using a BCA Protein Assay Kit (Pierce Biotechnology). Protein samples (80 mg) were denatured at 95°C and subsequently separated by SDS-PAGE gel electrophoresis. After being transferred to nitrocellulose, the membrane was blocked with 1% BSA, and the samples were probed with primary antibody, followed by a horseradish peroxidase-coupled (HRP) secondary antibody. β-Tubulin antibody was used as a loading control. A complete list of the antibodies used is included in Supplemental Table S1.

### CD44 antibody

The hybridoma (ref. ATCC PTA-9008) derived CD44-specific monoclonal antibody was purchased from ATCC (France) and the mAbs were purified on protein G-sepharose (Pierce Chemical). Reagents used for *in vivo* treatment were diluted in PBS and sterilized using a sterile 0.22 μm pore-size filter.

### Human PDAC samples

Tumor samples from 2 PDAC patients, treated with gemcitabine, were obtained by surgery, before and after treatment. A further 15 PDAC samples were obtained following surgery of untreated or chemotherapy treated patients, from the Pathology Department of the Hôpital Nord and Institut Paoli Calmettes, Marseille, France. All samples were embedded in paraffin and immunohistochemistry staining was performed on 5-μm sections, using the anti-CD44 antibody (BioLegends), following standard procedures.

### Flow cytometry

To characterize pancreatic cancer stem cells, the following antibodies were used: anti-CD44-APC, anti-EpCAM-VioBlue, anti-CD24-PE (MACS, Miltenyi Biotec) or appropriate isotype-matched control antibodies, and the ALDEFLUOR-FITC (ALDH-FITC) assay (Stem Cell Technologies, Vancouver, CA). For the ALDEFLUOR reaction, cells were diluted in ALDEFLUOR reaction buffer at a concentration of 1×10^6^ cells per ml. Controls contained both ALDH reagent and the inhibitor diethylaminobenzaldehyde (DEAB). Another set of controls contained ALDEFLOUR alone. The reactions were incubated at 37°C for 1 h. To set the gates for FACS analysis, one control had no ALDEFLUOR or antibody staining. Another control contained either secondary antibody staining alone or ALDEFLUOR+DEAB to set the gates for nonspecific staining. Samples were analyzed by flow cytometry, using a MACSQuant VYB flow cytometer (Miltenyi Biotec), and data were analyzed with FlowJo 9.4.4 (Treestar, Ashland, Oregon).

### Cell culture

For *in vitro* studies, tumor fragments were enzymatically digested with collagenase type V (Sigma) and trypsin/EDTA (Gibco, Life Technologies) and suspended in DMEM, supplemented with 1% (w/w) Penicillin/Streptomycin (Gibco, Life Technologies) and 10% Fetal Bovine Serum (Lonza). After centrifugation, cells were re-suspended in Serum Free Ductal Media (SFD), adapted from Schreiber et al. [43], at 37°C in a 5% CO_2_ incubator. Cells were weaned from antibiotics at least 48 h before performing tests.

### Chemograms

Cell chemosensitivity was assessed using 5 drugs commonly used to treat patients with PDAC, namely gemcitabine (Lilly), 5-fluorouracil (5FU) (Teva Pharma), oxaliplatin (Hospira), docetaxel (TXT) (Sanofi-Aventis), and an irinotecan active metabolite, 7-Ethyl 10-Hydroxycamptothecin or SN-38 (Sigma Aldrich). Five thousand cells per well were plated in 96-wells plates in SFD media. Twenty four hours later the media was supplemented with increasing concentrations of drugs (0 to 1000 μM and 0 to 100 μM for SN-38), and incubated for an additional 72 h period. Each experiment was done in triplicate and repeated at least two times. Cell viability was estimated after incubation with the PrestoBlue reagent (Life Technologies) for 3 h, following the PrestoBlue cell viability reagent protocol provided by the supplier.

### Statistical analysis

Results for continuous variables are expressed as means ± standard error of the mean (SEM). Overall comparisons of continuous variables were performed using the unpaired two-tailed Student's t-test, and non-normal distribution, unpaired data were assessed using the Mann-Whitney test. All tests of significance were two-tailed and the level of significance was set at 0.05. All data are representative of at least two independent experiments.

## SUPPLEMENTARY MATERIAL, FIGURES AND TABLES


