# Supplementary material for: Deciphering the cellular source of tumor relapse identifies CD44 as a major therapeutic target in pancreatic adenocarcinoma
Source: Oncotarget. 2015 Mar 10;6(10):7408–23. doi: 10.18632/oncotarget.3510 (PMC4480689; doi:10.18632/oncotarget.3510)
Supplement: Supplementary file 1 [file oncotarget-06-7408-s001.pdf]

Deciphering the cellular source of tumor relapse identifies CD44 as a major therapeutic target in pancreatic adenocarcinoma

Supplementary Material

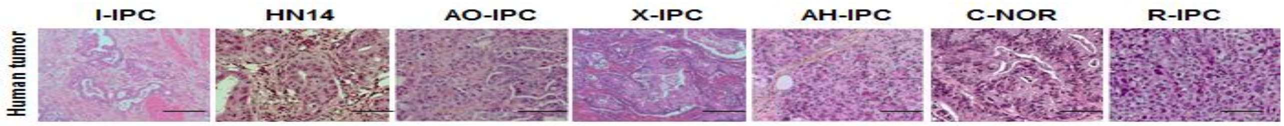

Figure S1: Examples of H&E staining of the PDACs obtained from human tumors. Scale bar: 100 µm.

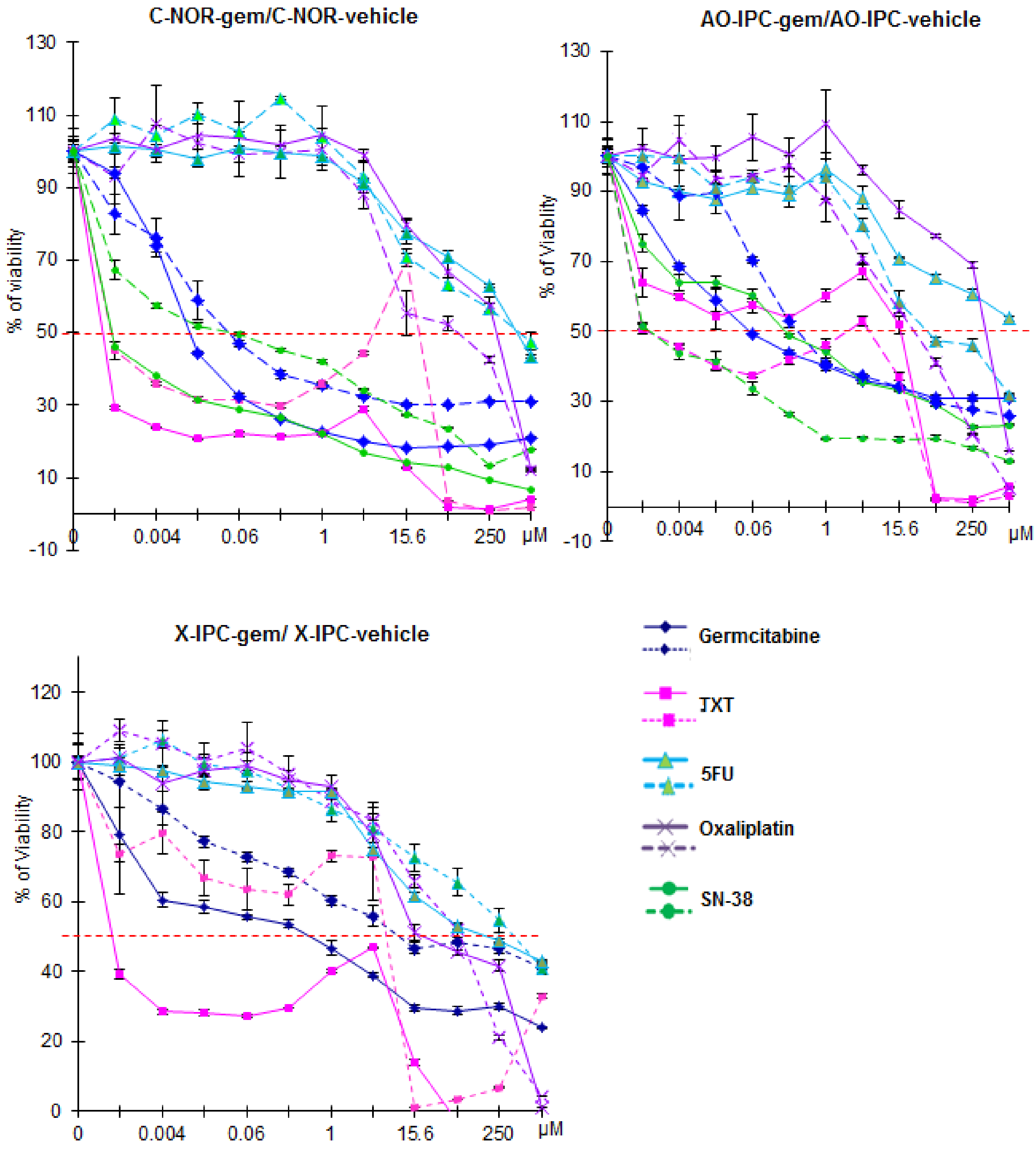

Figure S2: Representative curves for the chemogram analysis performed on xenograft-derived cells. The continuous line indicates vehicle-treated xenografts, and the discontinuous line shows curves for the gemcitabine-treated xenografts.

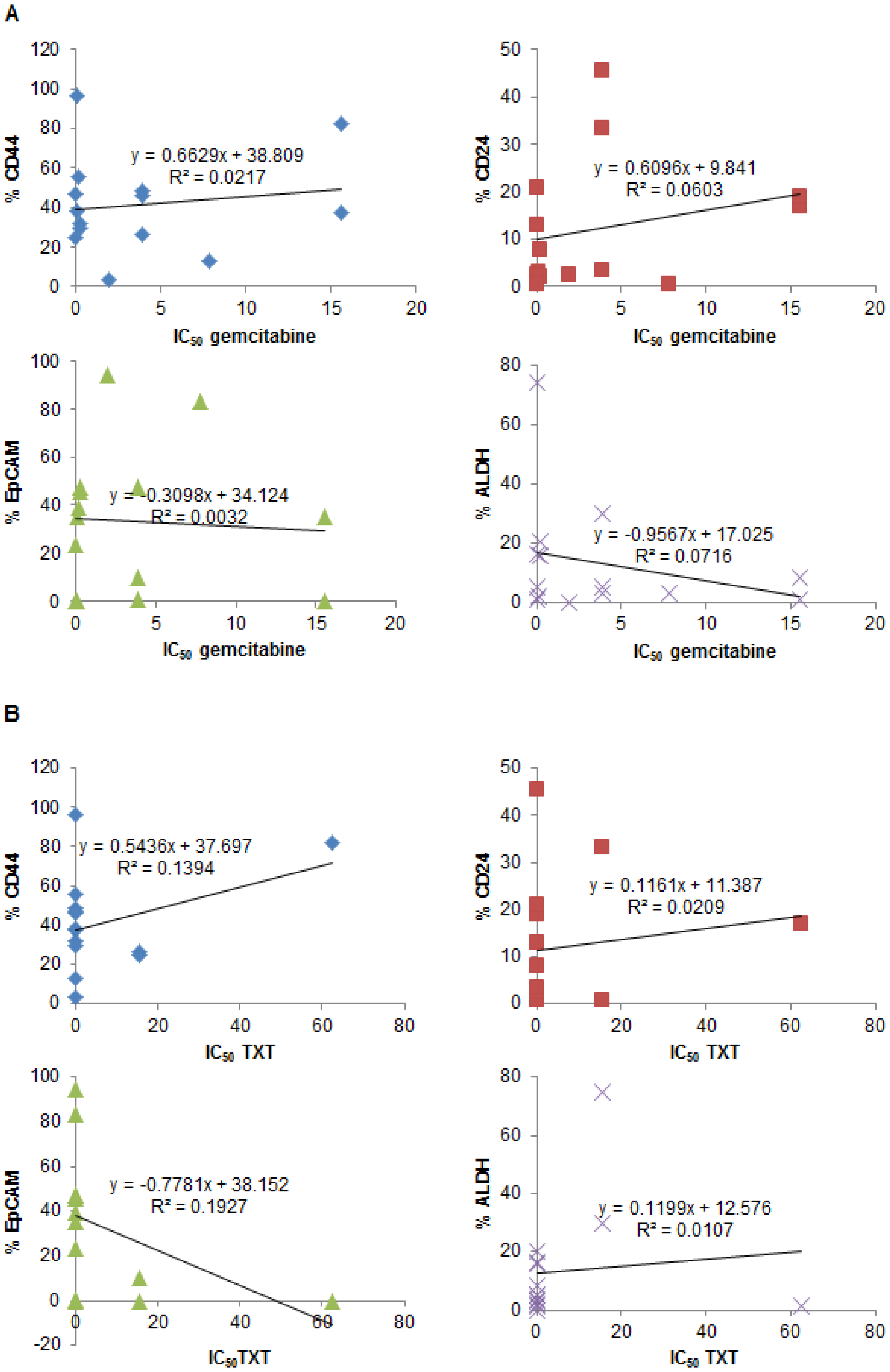

A
% CD44
IC50 gemcitabine
y = 0.6629x + 38.809
R² = 0.0217
% CD24
IC50 gemcitabine
y = 0.6096x + 9.841
R² = 0.0603
% EpCAM
IC50 gemcitabine
y = -0.3098x + 34.124
R² = 0.0032
% ALDH
IC50 gemcitabine
y = -0.9567x + 17.025
R² = 0.0716
B
% CD44
IC50 TXT
y = 0.5436x + 37.697
R² = 0.1394
% CD24
IC50 TXT
y = 0.1161x + 11.387
R² = 0.0209
% EpCAM
IC50TXT
y = -0.7781x + 38.152
R² = 0.1927
% ALDH
IC50 TXT
y = 0.1199x + 12.576
R² = 0.0107

C

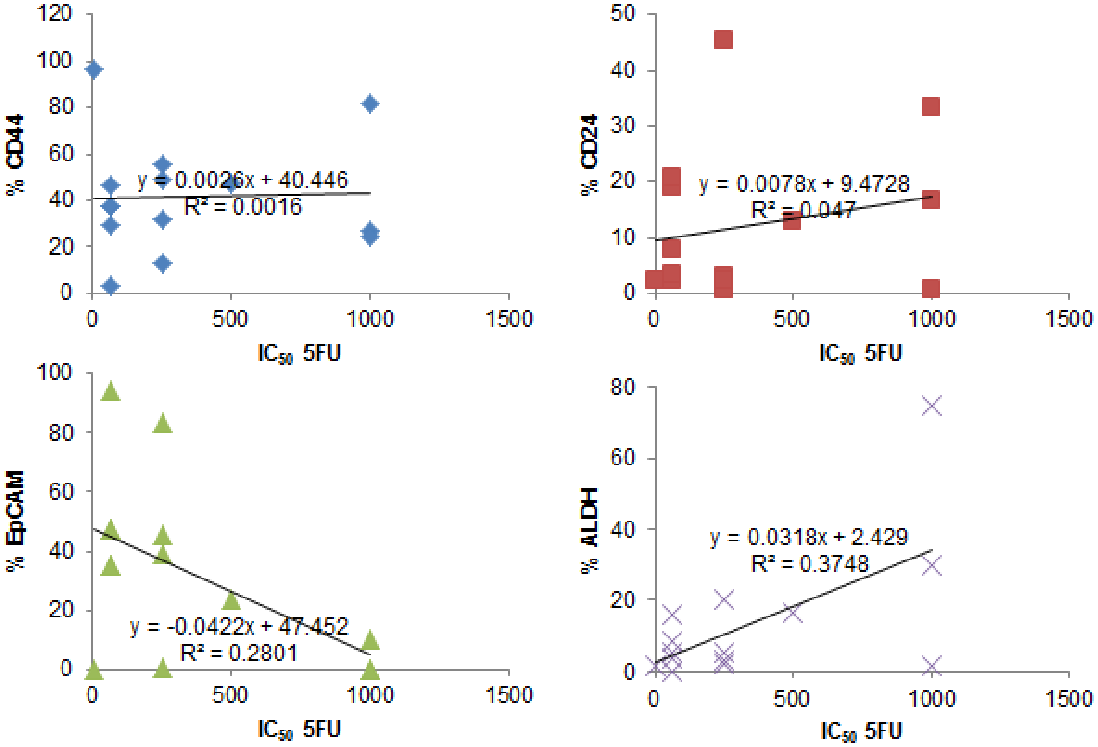

% CD44
y = 0.0026x + 40.446
R² = 0.0016
IC50 5FU
% CD24
y = 0.0078x + 9.4728
R² = 0.047
IC50 5FU
% EpCAM
y = -0.0422x + 47.452
R² = 0.2801
IC50 5FU
% ALDH
y = 0.0318x + 2.429
R² = 0.3748
IC50 5FU

D

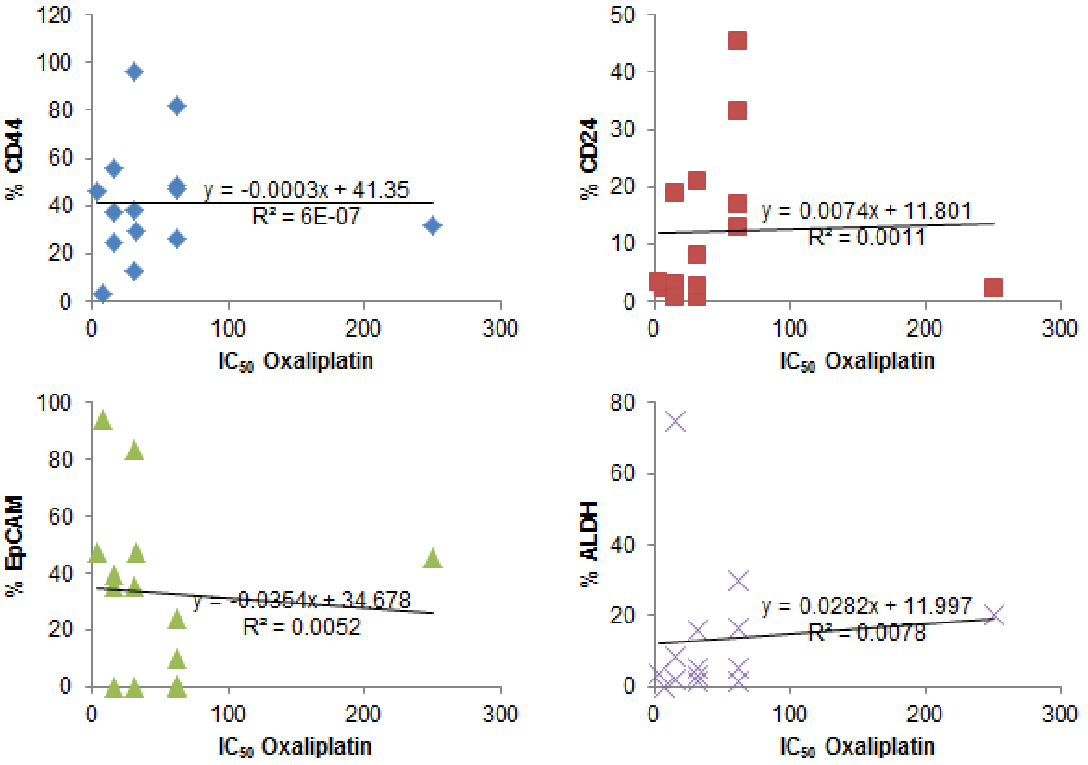

% CD44
y = -0.0003x + 41.35
R² = 6E-07
IC50 Oxaliplatin
% CD24
y = 0.0074x + 11.801
R² = 0.0011
IC50 Oxaliplatin
% EpCAM
y = -0.0354x + 34.678
R² = 0.0052
IC50 Oxaliplatin
% ALDH
y = 0.0282x + 11.997
R² = 0.0078
IC50 Oxaliplatin

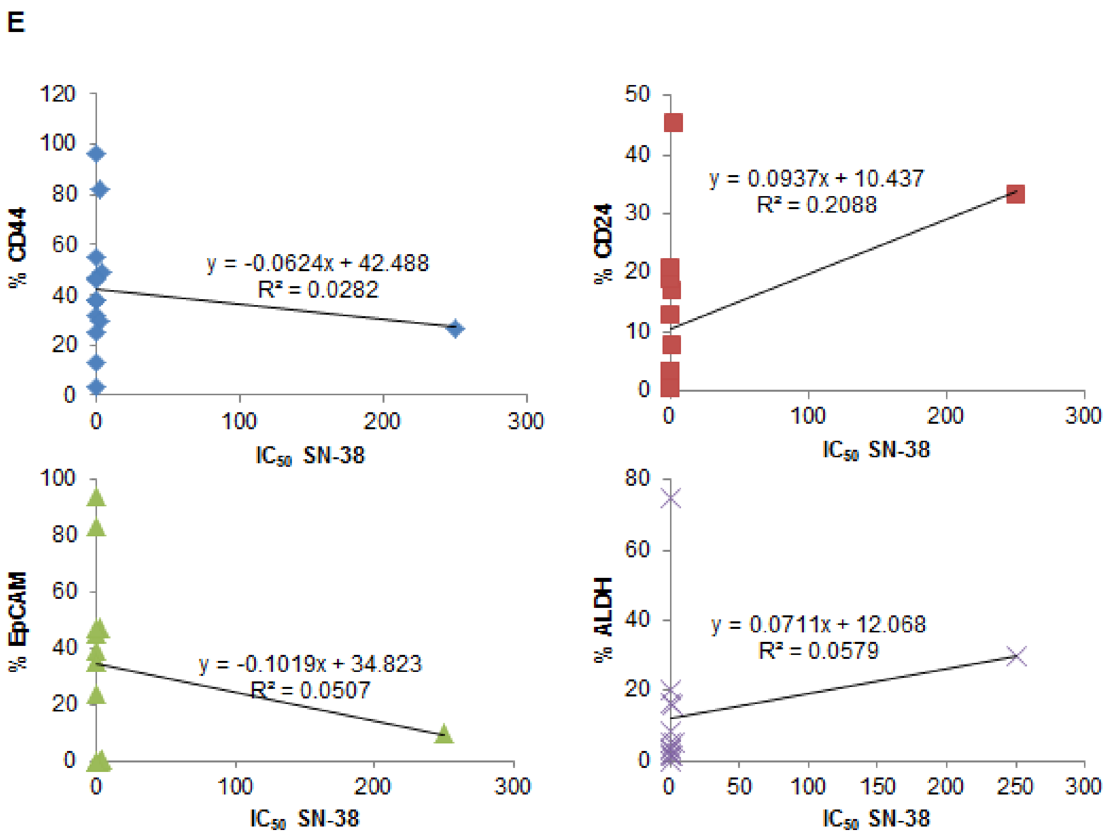

Figure S3: Linear regression of the mean values corresponding to CSC-marker expression against the IC_{50} of the anticancer drugs (A-E) for each cell line.

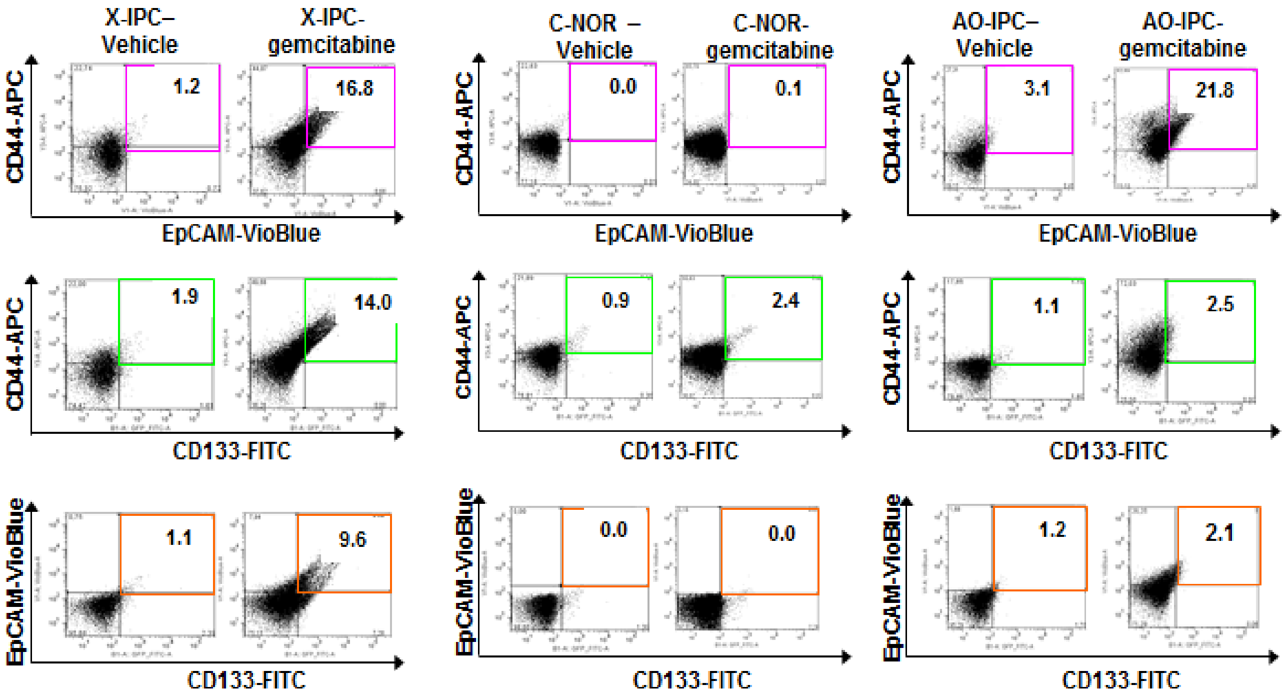

Figure S4: Flow cytometry of CD44-APC, EpCAM-VioBlue and CD133-FITC was used to analyze double positive cells. Representative plots are shown for each tumor.

Table S1: List of antibodies utilized for IF and Western Blotting.

Antibody	Source	Dilution	Manufacturer	Technique
CD44-FITC	Goat	1/400	Biolegendes	IF
CD133-FITC	Mouse	1/200	Merck Milipore	IF
EpCAM-FITC	Goat	1/400	Biolegendes	IF
CD44-APC	Mouse	1/50	Miltenyi Biotec	FC
CD24-PE	Mouse	1/50	Miltenyi Biotec	FC
EpCAM-VioBlue	Mouse	1/50	Miltenyi Biotec	FC
ALDH-FITC	Mouse	1/50	Stem Cell Technologies	FC
β-Tubulin	Mouse	1/1000	Invitrogen	WB
ZEB1	Rabbit	1/400	Santa Cruz	WB and IFI
MUC1	Mouse	1/500	Sigma	WB
MUC1	Rabbit	1/200	ABCAM	IFI

APC, allophycocyanin; FITC, fluorescence isothiocyanate; PE, phycoerythrin; IF, immunofluorescence; IFI, indirect immunofluorescence; WB, western blot; FC, flow cytometry

Table S2: The chief clinical characteristics of the patients studied and the principal biological information available for these tumors.

Tumor Name	Origin	Diagnosis	State/Differentiation	Tumor Size	Treatment in humans	Recurence	Passage in Mice
R-IPC	Biopsie	Metastasis	Moderately	T4	Folfirinox + RDT	NA	6
I-IPC	Surgery	ADK	Moderately	T3	Gemcitabine	Yes	5
AO-IPC	Surgery	ADK	Moderately	T3	Gemcitabine	Yes	5
AH-IPC	Surgery	ADK	Moderately	T3	Gemcitabine	Yes	6
X-IPC	Surgery	ADK	Poorly	T4	Gemcitabine	Yes	5
C-NOR	Surgery	ADK	Well	T3	Gemcitabine	Yes	5
HN14	Biopsie	ADK	Moderately	T4	Folfirinox	Yes	5

Table S3: Expression of CD44-APC, CD24-PE, EpCAM-VioBlue and ALDH-FITC markers in 14 different primary cell lines**.** Error bars ± SEM; n=3 per group.

	CD44		CD24		EpCAM		ALDH	
	Mean	SEM	Mean	SEM	Mean	SEM	Mean	SEM
**Foie-8b**	46.1	0.9	3.3	0.2	47.2	0.5	3.4	0.1
**01.030**	24.8	1.1	0.5	0.3	0	0	74.5	1.2
**HN-01**	47	0.8	12.9	0.7	23.5	0.3	16.4	0.3
**E-NOR**	29.1	3	7.8	0.6	47.2	1.6	15.7	0.9
**AH-IPC**	31.8	1.2	2.2	0.7	45.4	0.8	20.4	0.3
**AD-IPC**	48.6	1.5	45.3	0.5	0.6	0.5	5.3	0.6
**L-IPC**	96.2	1.2	2.5	0.6	0	1.7	1.2	0.31
**J-IPC**	37.9	1.55	20.8	0.9	35.4	0.78	5.4	0.42
**D-IPC**	26.6	1.2	33.3	0.7	10	0.8	30	3.5
**B-TIM**	55.3	2.5	3.1	0.4	39.3	1.4	2.1	0.1
**A-NOR**	3.2	0.5	2.4	0.4	94.2	2.5	0	0
**HN-03**	37.4	1.5	18.9	0.8	35	1	8.5	0.5
**H-NOR**	12.8	1.7	0.5	0.4	83.4	1.5	3.2	0.2
**01.001**	81.9	3.5	16.8	2.3	0	0.2	1.2	0.3
